# Senility, Senile Dementia and Their Medicolegal Aspects1Read before the Syracuse Academy of Medicine, December, 1900.

**Published:** 1901-03

**Authors:** F. H. Stephenson

**Affiliations:** Syracuse, N.Y., Neurologist to Syracuse Hospital for Women and Children


### SENILITY, SENILE DEMENTIA AND THEIR MEDICO-LEGAL ASPECTS[1].

By F. H. STEPHENSON, M. D., Syracuse, N.Y.,
Neurologist to Syracuse Hospital for Women and Children.

WHEN using the term senile or senility we refer to advanced age, but from a medical standpoint the number of years a man has lived has often little bearing, anatomically and physiologically, for many from forty-five to sixty years of age show the symptoms of degeneration or presenility, which changes should normally occur about the seventh decade of life, when general involution of the whole body begins with loss of functional activity of the brain.

How true, pathologically, is the saying "man is as old as his arteries," and how frequent the symptoms of degeneration following these circulatory diseases. Forgetfulness and insomnia are often marked in mankind after fifty years of age, regardless of other mental infirmities. Physical symptoms of senility usually precede mental changes and consist of general decrepitude, tremor of the hands, facial muscles and tongue, and the stigmata of focal brain disturbances, such as aphasias or monoplegias. These may be temporary, due to circulatory disturbances, or permanent, when due to aneurisms, embolisms or hemorrhages.

---

1. Read before the Syracuse Academy of Medicine, December, 1900.

Case I.—Mrs. M., aged 76 years; presents quite a typical history. She complains of intense vertigo, distress in the back of her head, ringing in her ears, insomnia, paraesthesia and hyperesthesia, sparks, lines and flashes of red and yellow light before the eyes; there are vaso-motor symptoms, such as hot flashes and drenching perspiration. Quite recently there have been observed progressive mental enfeeblement, sensitiveness, confusion, impatience.

These patients show an inability to recall dates, are subject to vacillation, become easily disturbed, yield readily to the entreaties of their children or friends, make foolish marriages and unjust wills. They transfer their property unwisely; their memory for recent events is very unstable, they forget the events of yesterday, but give most accurate and interesting data of their earlier lives, the development of the country, its prominent men, their sayings; yet while relating these matters they forget who you are, your errand, and cannot tell the day of the week, or the month of the year. Their physical comforts often are all they care for, they forget family and friends, and call their children by other than their own names. Memory and judgment associations have failed, and sad to relate, they commit many breaches of decorum. Though living in fine homes they imagine poverty is staring them in the face, and they almost starve themselves, and sometimes reek in filth; they develop delusions of persecutions, grow melancholy or excited, wander about at night and keep an entire family or institution in an uproar. With these more marked changes from the normal the patient has developed a complete picture of insanity, with all of its moods and fancies, the usual form, history and development being that of senile dementia, its features and delusions being characteristic.

Senile insanities may manifest themselves in manias, melancholias, incoherent paranoias and dementia, all modified by weakened cortical function with special delusions, but particularly those of coming poverty or want.

These demented old people are often very troublesome and have to be committed to hospitals for the insane. The regularity and restraint of an institution often acts beneficially and they spend their days in greater comfort, though many realise their confinement, and considering it unjust, they make a great disturbance for all about them and seem the most trying of all lunatics.

ETIOLOGY.

*Heredity.*—Mental strain, physical illnesses, arterio-sclerosis, focal degeneration, trophic changes, cerebral hemorrhage, general marasmus of nervous tissue, enfeeblement due to age, causing an organic psychosis, all appear to be the conditions developing this state of mind.

*Diagnosis.*—General enfeeblement, defective memory and judgment, loss of ethical feeling, a changed *ego*, lessened sympathies, slow perception and thought, arcus senilis, hardened radials and tortuous temporals, are evidences of mental and physical decay or degeneration.

*Prognosis.* —Remissions in some symptoms occur in these cases, but those well advanced in years with marked dementia never recover. They may have capacity for receiving impressions, but they cannot formulate ideas. Should no intercurrent disease occur to cause death, they drift on from bad to worse, developing bed sores, diarrhea, cystitis, colliquative sweats and finally death.

*Pathology.*—We find the brain diminished in weight in many cases, distension of the ventricles and vessels, signs of atheroma, pachymeningitis hemorrhagica, degeneration of ganglion cells and fibers, encepholamacia, sclerotic hardening and hemorrhages. An increase of hemoglobin and leucocytes often occur.

The lines of sanity and insanity are very closely drawn, yet they vary with different alienists and psychologists. Many men only regard as insane any act which is positively wrong; other deviations they call eccentricities. Many alienists claim a man insane who is lacking in memory, judgment, the power of concentration and will. One must weigh these standards of opinion carefully, for while the former draws his fine lines of diagnosis and acts by them, circumstances may present themselves whereby another may be as accurate and have weighed all of the physical and mental symptoms, yet have his lines of diagnosis drawn at a different standard. Many men have mental ability and are truly geniuses, but lack in mental stability and earlier or later lose balance and go over the so-called normal line of sanity. As physicians we have to deal with the testamentary capacity and the sequestrations of the persons and property of these old people.

The family physician, who has been in attendance for years should recognise the changed *ego*, manner, custom, habits, and thus form an opinion of a person's sanity. Reports given us must be weighed carefully. At first they may appear delusions, when on further investigation we find them true occurrences, also regarding their care and the management of property, and whether the one who applies for a commission to be appointed is interested financially, or is it a matter solely of philanthropy?

Some attorneys claim we examine these patients or testify on the witness stand, after having heard the hypothetical questions, with preconceived opinions. This cannot be helped, as we must have the previous history before arriving at a conclusion. Experts know too well the unreliability alone of a personal examination. It is the past life and the many factors put together which make the diagnosis even without delusions. It must be remembered they often extend beyond the form in which they manifest themselves, and may influence the mind in important matters. We think with Blanford, who said: "It is always satisfactory to find delusions for they indicate a stage of insanity when perverted feelings and emotions have taken the shape and form of insane ideas and conceptions."

Lord Brougham, quoted by Hamilton, in Nervous and Mental Jurisprudence, said: "A mind which labors under a certain delusion, though apparently sane on all other points is not so—it is only sound in appearance."

A medical man well experienced should be able to recognise a deranged mind, and to know if such a state of derangement is sufficient to account for one's actions. Look to the grade of his mental power, to the notion by which he is controlled, to his views of matters in general, tot he past course of his life, and as Ordroneaux said, "look to his power of control over his doings or actions, whether this power is regulated or running at random.

We must remember insanities, like most of the neuroses, are characterised by exacerbations and remissions, thus explaining the great variance in the patient's ideas and movements.

Case II.—Comprises a will executed by a childless widow, 68 years of age, disposing of over $30,000. Her first bequest was to the son of her former coachman, not at all related to, nor having any claim upon her. The second, to an agent who had been well paid monthly for his labors and the third to a former partner of her late husband, who had for some years kept them both in a state of unrest and fear; while her near relatives, in whom she had always been interested, and who had frequently been told her property would descend to them, received absolutely nothing. Imagine the person making this will having lived in excellent style, having servants, horses, carriages and other luxuries, beside having a fine social position, changing to such an extent that she disposed of all luxúries and nearly all necesities, attending to all of her household duties, having very meager meals because she could afford but little food, sitting in a cold room, as she could not afford a fire, dressing poorly and untidily, having increasing deafness, with attendant suspicion, in fact exhibiting a changed manner of life and *ego*.

Could such a person make a just will which would be according to her intention when in normal health? The affirmative would say, "she might have received such treatment from relatives as would cause her to change her plans, or, her new friends she may have considered more worthy, and more needy. Again, the affirmative might say her income was so much less than in earlier life that her later income seemed paltry in comparison, and this accounts for her changed manner of living. Elderly people usually grow penurious and have less expensive habits than in earlier life, but from the history given, marked signs of physical decay, with palor, tremor, increasing deafness, suspicions, feebleness, ideas of poverty and fear, I believe our picture to be one of senile dementia, and that a will executed by such a person would doubtless be unjust.

Case III.—A man, aged 80 years, presented a picture of senile infirmity. He forgot the name and number of his children, as well as the towns in which they lived. He had no memory of later or recent events, became confused regarding the late Civil War and the recent Spanish Wars. One child has managed to have certain bank deposits transferred to her account, while other of his children, who are far more needy, and the ones on whom he depends for daily care, could not prevent this injustice.

We certainly can not expect such a person to make a just disposition of his estate, yet such persons are constantly defended by our best attorneys in surrogate and other courts of justice. Occasionally, we meet cases where some special delusion is the marked feature, other usual indications of dementia not being well marked. In such cases a will might be accepted, provided the delusion was not of such a character as to influence the executor of the will at all regarding his pecuniary affairs. This is a changed idea from that which was accepted years ago. It is an American and English law, but not accepted by all alienists, who claim man has one and the same mind for all actions dependent upon reason and judgment, and if he cannot reason the delusion away his judgment is impaired and therefore he is unfit to transact business.

The laws of science cannot always decide the care of these senile dements; facts and common sense must decide very often their needs, and the natural care of their person and properties.

---

Pain in the lower limbs, or the slightest degree of limping in children, should lead to an examination of the hip-joints. Many cases of beginning hip-joint disease may be discovered at a time most opportune for treatment.—*Interstate Medical Journal.*

